# Consumer willingness to pay for green express packaging in e-commerce: an eye-tracking experiment analysis

**DOI:** 10.3389/fpsyg.2025.1615315

**Published:** 2025-07-22

**Authors:** Zhenshui Yan, Tianbo Wang, Zizheng Song, Jiarui Liu, Wei Lyu

**Affiliations:** ^1^School of Economics and Management, Shenyang Aerospace University, Shenyang, China; ^2^School of Economics and Management, Anhui Polytechnic University, Wuhu, China

**Keywords:** green express packaging, willingness to pay, eye-tracking experiment, price sensitivity, environmental awareness

## Abstract

With the rapid development of e-commerce, packaging waste has surged, making green express packaging (GEP) a key component of sustainable logistics promotion. This study investigates the influence of product types (search goods vs. experienced goods), price sensitivity, and environmental awareness on consumers’ willingness to pay for GEP. Data were collected through a combination of questionnaire surveys and eye-tracking experiments, and analyzed by SPSS 27.0. The results show that consumers with higher environmental awareness demonstrate a greater willingness to pay for GEP premiums, particularly when purchasing experienced products. However, price plays a significant restrictive role in the choice of green express. Eye-tracking data reveal that green express option attracts more visual attention than traditional one. Although higher premiums reduce willingness to pay, they do not diminish visual attention. E-commerce platforms and express delivery companies should consider product type, consumers’ environmental awareness, and price sensitivity when formulating pricing strategies for green packaging to optimize consumers’ willingness to pay.

## 1 Introduction

As an indispensable part of e-commerce, express delivery industry has shown explosive growth in recent years, with the rapid development of online shopping. According to statistics from the State Post Bureau of The People’s Republic of China (2025), China’s express delivery volume has reached 132.07 billion in 2024. However, the rapid increase in delivery volume has led to an exponential increase in packaging waste, resulting in numerous negative impacts (Nguyen Quoc et al., 2025).

To address this challenge, China has explicitly outlined the goal of “promoting the development of green logistics, particularly advancing the application of green packaging” in the “14th Five-Year Plan” for Modern Logistics Development (General Office of the State Council of The People’s Republic of China, 2022). Green packaging is a form of packaging that achieves resource conservation and minimizes environmental harm as much as possible during its life cycle (Yan and Lin, 2011; Isa and Yao, 2013). Addressing the issue of express packaging waste and promoting green packaging application requires the joint efforts of the government, enterprises, and consumers. Consumers need to adjust their environmental consciousness and behavior (Chen, 2016). Although the improvement of socio-economic levels has significantly increased consumers’ attention to green packaging (Jiang, 2023), it often comes with a higher price and requires consumers to pay the premium (Wandosell et al., 2021). Green express packaging (GEP) specifically for logistics and delivery utilizes recyclable or biodegradable materials and minimalistic designs to reduce waste and resource consumption. While sharing the same principles as green packaging, it focuses on minimizing environmental impact during transportation and delivery.

Some studies have pointed out that price is one of the key factors influencing consumers’ willingness to purchase green products (Hao et al., 2019). Consumers’ willingness to pay for green products is negatively correlated with the premium (Zhao et al., 2015), especially for price-sensitive consumer groups (Teoh et al., 2022). In China, although consumers are willing to consume green products, most consumers have not realized actual purchasing behavior for various reasons (Zhang and Li, 2017), product price is one of the major influencing factors (Li et al., 2016). Research based on data collected from field surveys indicates that most consumers are willing to pay a premium for green products, but their willingness to pay varies with different premium levels (Sheng et al., 2018), Another recent study suggests that consumers’ environmental awareness also plays a crucial role in their purchasing decisions (De Canio, 2023). A survey study targeting Chinese consumers found that approximately 70% of residents in first-tier cities, where environmental awareness tends to be higher, are willing to pay ¥2.46 for GEP (Lin and Wang, 2023). Another study, which collected consumer data from randomly selected second- and third-tier cities through field surveys, found that consumers generally have a willingness to use and pay for GEP with additional ¥2.18 (Jia et al., 2025). Although consumers in these lower-tier cities are also inclined to choose GEP, differences in environmental awareness can be attributed to one of the factors of their lower willingness to pay when compared to their counterparts in first-tier cities. In e-commerce, where consumers encounter various options and prices, environmental awareness becomes a pivotal factor in balancing cost and sustainability in their decisions. Previous research statistic data on GEP premiums primarily relies on questionnaire surveys, with limited research on visual attention. Survey data tends to be subjective and lacks the real-time capture of consumers’ decision-making process, which can affect the objectivity and accuracy of the results. Eye-tracking technology can help address these shortcomings to some extent.

This paper aims to explore consumers’ willingness to pay for GEP on e-commerce platforms at different premium levels. By combining questionnaire survey and eye-tracking experiment, this paper examines consumers’ visual attention and decision-making processes when facing express delivery options with different premiums on the online shopping checkout interface. Additionally, this paper provides empirical evidence to help e-commerce platforms and express delivery companies formulate pricing strategies for GEP, offering guidance and recommendations for future optimization plans.

## 2 Literature review and hypothesis development

### 2.1 Factors affecting the willingness to pay for GEP

In addition to price, the type of products also plays an important role in consumers’ willingness to pay. The distinction between search goods and experience goods, proposed by Nelson, is an important classification in business marketing (Nelson, 1970). Search products are products whose quality and value can be assessed by consumers through prior information gathering, while experience products require consumers to experience them firsthand in order to perceive their quality (Eathers et al., 2007). The increased detail and completeness of online product reviews have significantly enhanced consumers’ perceptions of products, making them more price-sensitive to search products. As a result, their willingness to pay for green express delivery premiums may be more strongly influenced by price. In contrast, for experience products, consumers may be more inclined to accept the added value provided by green packaging, showing a higher willingness to pay (Zhao and Han, 2015; Wang et al., 2016). Consequently, the following hypotheses are proposed:

*Hypothesis 1.* Consumers have a higher selection rate for green express option when purchasing experience products compared to search products.

### 2.2 Eye-tracking research method

Unlike traditional survey methods that rely on self-reported preferences, eye-tracking relies solely on physiological data and enables marketers to understand consumers’ cognitive engagement and tailor information to create effective marketing strategies (Duchowski, 2007). This technology allows researchers to capture real-time, objective data on consumers’ visual attention during decision-making processes. By tracking the subject’s gaze position and duration using advanced equipment (Zheng et al., 2022), it provides a clear understanding of what the subjects focus on, how they engage with the stimuli, and the duration of their attention on stimuli (Lin and Yang, 2014). Additionally, it would strengthen the methodological background to briefly highlight that eye movements serve as an objective measure of visual attention, as demonstrated in earlier cognitive research. As e-commerce has become an essential part of modern retail, the application of eye tracking in this field has increased significantly, especially in research related to consumer behavior reactions on online reviews. Previous researches have extracted indicators from review data to identify the factors that influence review quality (Otterbacher, 2009). Subsequent articles provide a detailed analysis of factors such as font size (Huang, 2025) and word count (Ghose and Ipeirotis, 2011). Other researches are based on the review information from web product interfaces as material for eye-tracking experiments, collecting consumer feedback on different types of products and review formats (Diao et al., 2017; Wang et al., 2020) and providing relevant suggestions for businesses to optimize product page information (Tang, 2020). The application of eye-tracking technology in online reviews has proven the feasibility of using web information as experimental material. Consequently, the following hypotheses are proposed:

*Hypothesis 2.* Consumers' fixation time on green express options varies depending on the type of product.

*Hypothesis 3.* The premium level has a negative effect on the willingness to pay, and the product type moderates this relationship.

### 2.3 Theory of planned behavior

The theory of planned behavior (TPB) is developed based on the theory of reasoned action (TRA). TRA believes that behavioral attitudes and subjective norms directly affect behavioral intention and behavioral intention directly determine actual behavior (Yan, 2014). TPB adds perceived behavior control on the basis of TRA. Consumers’ purchasing behavior is influenced not only by their purchasing attitude but also by the impact of society and ones around them, and is also constrained by factors such as personal purchasing power, opportunities, and various resources (Yang et al., 2018). Previous research found that consumer attitudes and perceived behavioral control can positively drive consumers’ willingness to purchase and behavior toward green products (Zhang, 2021). Another research showed that consumers’ environmental awareness can positively drive green consumption actions, while regional economic level and environmental quality have a significant impact on green consumption willingness (Ural and Zeng, 2023). The eye-tracking methodology used in this study provides valuable insights into how consumers form attitudes and intentions by analyzing their visual engagement with green express packaging options. By combining eye-tracking data with the TPB framework, we can explore the cognitive and behavioral factors that influence consumers’ willingness to pay for green packaging in e-commerce.

In terms of GEP, consumers’ willingness to accept GEP has been demonstrate to be directly driven by perceived usefulness, perceived behavioral control, and environmental responsibility awareness (Pan and Guo, 2019). In the context of choosing GEP for express delivery, attitude refers to consumers’ overall evaluation and perception of GEP based on past green experiences and cognition; subjective norms refer to the influence from external sources (others, society, etc.) when making decisions; perceived behavioral control refers to consumers’ subjective judgment about whether they are capable of paying for the price. Consumers obtain information about GEP from personal experience and environmental observation, and they make actual payment behavior after evaluating its price and their own economic situation (Le and Nguyen, 2022; Sheng et al., 2024). Consequently, the following hypotheses are proposed:

*Hypothesis 4.* The higher the consumer's environmental awareness, the more willing they are to pay the extra cost for GEP when faced with an additional premium for GEP.

## 3 Methodology

A questionnaire survey and eye-tracking experiment were implemented to analyze participants’ subjective and non-subjective responses to our questions and stimuli.

### 3.1 Participants

College students frequently participate in and rely on online shopping and e-commerce platforms. Related research indicates that college students’ consumption behavior is relatively flexible, but due to limited income sources (usually relying on family support or part-time earnings), they exhibit high price sensitivity when choosing products (Li et al., 2017). Their willingness to pay for GEP is often influenced by budget constraints when facing the premium. In this paper, 65 college students (37 males, 28 females) were recruited with online shopping experience from a college to participate in this eye-tracking experiment. All participants were right-handed and had normal vision or corrected-to-normal vision. They were assured that the information would be solely used for academic purposes and their responses would be aggregated to maintain anonymity. Due to the quality issues with the eye-tracking data, only 59 participants’ data out of the 65 participants were included in the final analysis. Data from the remaining 6 participants were excluded due to incomplete trials or significant technical issues such as excessive blinking or loss of gaze, which compromised the reliability of the data.

### 3.2 Experiment design and procedure

This experiment simulates a real online payment scenario based on the Taobao shopping checkout interface. To ensure that participants could clearly differentiate between two types of products, we conducted a manipulation check. Participants were given brief descriptions of both product categories and asked follow-up questions to verify their understanding. This check ensured that participants correctly categorized the products as either search or experience products before proceeding with the experiment. After browsing the products and checkout interface details participants chose the final express delivery method and whether to proceed with payment. Participants’ behavioral choice data (green express selection rate) and eye-tracking metrics data for each area of interest were collected.

**Figure 1:**
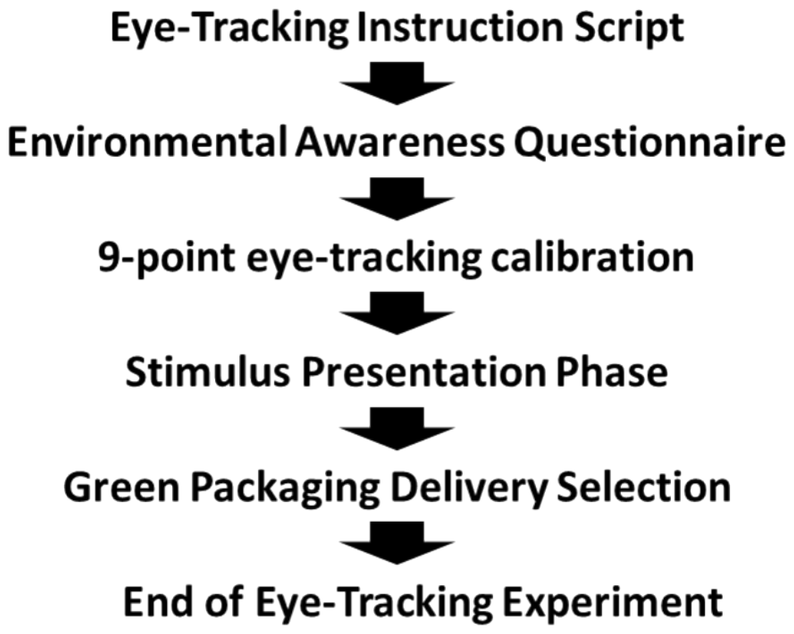
Eye-tracking choice experiment procedure.

The entire experiment lasted approximately 5 min and was divided into three stages. First, before the experiment began, participants were asked to sign a consent form and were informed of the procedure. Participants completed a questionnaire that collected basic information and assessed environmental awareness by a five-point Likert scale (adapted from Lin and Wang, 2023), A five-point Likert scale was used to assess participants’ level of agreement with statements ranging from 1 = Strongly Disagree to 5 = Strongly Agree (see Table 1). They were also informed of the definition of GEP. Before recording eye movements, a nine-point calibration procedure was conducted to adjust the participants’ visual attention. Participants were instructed to select while maintaining a distance of approximately 65 cm from the eye-tracking device. Second, the formal experiment proceeded as follows (see Figure 1): On a 24-inch computer screen with a resolution of 1,920 × 1,080 pixels, participants viewed 8 images representing two groups of product checkout scenarios. The first image of each product displayed the traditional express method, followed by 3 randomly presented images of green express options (different premium ¥1/¥2/¥5). Each image was displayed for 10 s to ensure participants had enough time to observe the scene. After viewing the four images in each product group, participants made a choice regarding the express delivery service. Finally, after the experiment ended, participants completed a supplementary questionnaire discussing the reasons for choosing green express and their acceptance of the premium, and received a cash reward of 20¥.

**Table 1:** Environmental awareness questions.

1. I care about environmental protection and sustainable development.
2. I believe green express helps protect the environment.
3. In daily life, I am willing to choose more environmentally friendly products or services, even if they are more expensive than regular ones.
4. I believe green express can effectively enhance citizens’ environmental awareness.
5. My concern for environmental protection influences my consumer decisions when shopping online.

**Figure 2:**
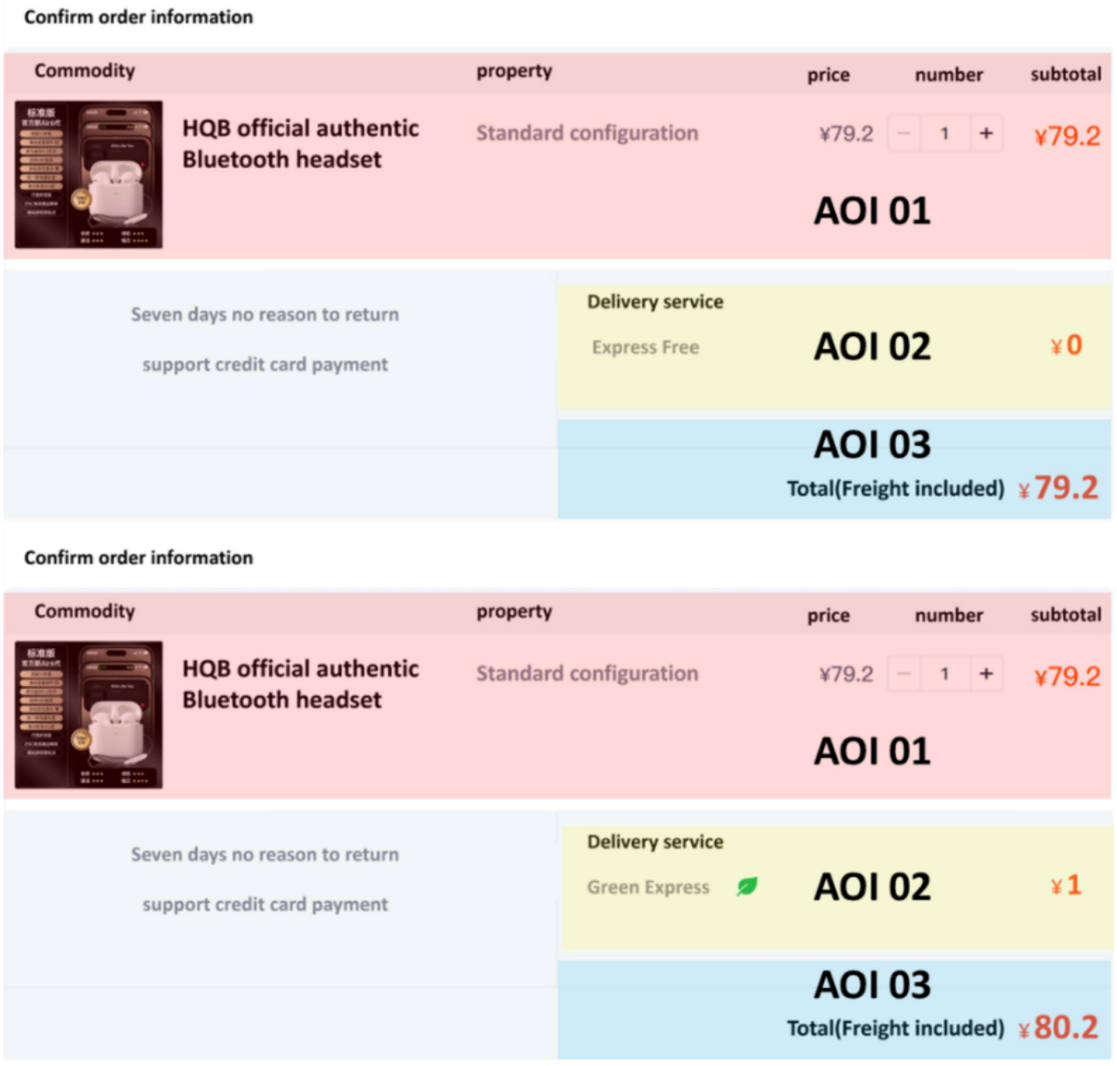
Samples of experimental materials.

To simulate a real online shopping checkout scenario as closely as possible, the commodity information and price selected for this experiment are based on real data from the Taobao website. Each experimental material is divided into three areas of interest: Commodity Parameter AOI 01, Delivery Method AOI 02, and Price Information AOI 03, as shown in Figure 2.

### 3.3 Data analysis applied

The eye tracking data were recorded with the Tobii X3-120 eye tracker and Tobii Pro Lab software. Data about fixation duration, number of fixations in the AOI, number of selections on GEP and heatmaps were then exported to be analyzed with the statistical software IBM SPSS Statistics 27. Statistical analysis included descriptive statistics to summarize eye-tracking metrics, mixed repeated measures ANOVA was conducted to assess the effects of product type and premium level on fixation duration for express options, correlation analysis was performed to examine the relationship between fixation duration and willingness to pay for GEP.

## 4 Results and discussion

### 4.1 Survey results analysis

In this paper, SPSS 27.0 was used to test the reliability of the questionnaires. The Cronbach’s alpha reliability coefficient is 0.816 > 0.8, suggesting that this questionnaire has a high credibility. The test coefficient of sample adequacy (Kaiser–Meyer–Olkin) is 0.785, which is greater than the experience value of 0.5. Bartlett’s spherical test chi-square approximation value is 81.820, the degree of freedom is 10, and the significance is 0.000, indicating that factor analysis is suitable for this study.

### 4.2 Behavioral choice data analysis

To verify whether different product types and premium levels affect participants’ choice rates for different delivery methods, this study conducted a 2 (Product Type: Search Products/Experience Products) × 3 (Packaging Premium: ¥1/¥2/¥5) mixed repeated measures ANOVA on the data regarding green express choices with different packaging premiums.

The results show that the main effect of premium level is significant (*F*(2,174) = 19.947, *p* < 0.001, *η*^2^ = 0.187). As show in Table 2, when participants faced low premium options, the choice rate for green express (*M*_S_ = 0.508, SE = 0.504; *M*_E_ = 0.763, SE = 0.429) was significantly higher than when they faced medium or high premium options (*M*_S2_ = 0.390, SE = 0.492; *M*_S5_ = 0.153, SE = 0.363; *M*_E2_ = 0.509, SE = 0.504; *M*_E5_ = 0.322, SE = 0.471). The main effect of product type is significant (*F*(1,174) = 14.878, *p* = <0.001, *η*^2^ = 0.079), with participants choosing green express for experience products at a significantly higher rate (*M*_1_ = 0.763, *M*_2_ = 0.509, *M*_5_ = 0.322) than for search products (*M*_1_ = 0.509, *M*_2_ = 0.390, *M*_5_ = 0.153). The interaction effect between product type and premium level is not significant (*F*(2,116) = 0.712, *p* = 0.492 > 0.050, *η*^2^ = 0.008). Overall, the green express choice rate for experience products is consistently higher than for search products, particularly at low premium levels, indicating that consumers are more willing to pay for environmental protection when purchasing experience products. Additionally, although higher premiums reduce participants’ willingness to choose, the decline for experience products is relatively gradual, while the decline for search products is steeper. However, no significant interaction effect was found between product type and premium level, suggesting that their effects are relatively independent. H1 is verified.

**Table 2:** Green selection rate.

Type	Price	Mean	SE
Search	¥1	0.509	0.504
	¥2	0.39	0.492
	¥5	0.153	0.363
Experience	¥1	0.763	0.429
	¥2	0.509	0.504
	¥5	0.322	0.471

Based on the environmental awareness scale items in the questionnaire survey, this paper scored the environmental awareness of all participants and divided them into three groups according to the median score: The low environmental awareness group (23 people with scores of 18 and below), the medium environmental awareness group (8 people with a score of 19), and the high environmental awareness group (28 people with scores of 20 and above).

A mixed repeated measures ANOVA was conducted, and the results showed in Table 3, the effect of environmental awareness was significant (*F*(1,98) = 6.167, *p* = 0.081 > 0.050). When faced with low-premium green express, the choice rate of green express among the low environmental awareness group (*M*_S_ = 0.391, *M*_E_ = 0.652) was significantly lower than that of the high environmental awareness group (*M*_S_ = 0.679, *M*_E_ = 0.821). When faced with medium-premium green express, the choice rate of green express among the low environmental awareness group (*M*_S_ = 0.304, *M*_E_ = 0.435) was also significantly lower than that of the high environmental awareness group (*M*_S_ = 0.429, *M*_E_ = 0.607). When faced with high-premium green express, the choice rate of green express among the low environmental awareness group (*M*_S_ = 0.087, *M*_E_ = 0.391) was still significantly lower than that of the high environmental awareness group (*M*_S_ = 0.143, *M*_E_ = 0.286). From the data, it can be seen that the high environmental awareness group showed a higher choice rate for green express under different premium conditions, indicating that they have a stronger willingness to pay when facing GEP premiums. However, when consumers with higher environmental awareness face higher premiums, their green express choice rate significantly decreases, suggesting that price sensitivity constrains their willingness to pay. H4 is verified.

**Table 3:** Environmental awareness (EA) selection rate.

EA	Type	¥1	¥2	¥5
Low	Search	0.391	0.304	0.087
	Experience	0.652	0.435	0.391
High	Search	0.679	0.429	0.143
	Experience	0.821	0.607	0.286

### 4.3 Eye tracking data analysis

Eye-tracking metrics can accurately reflect participants’ eye movement processes. Fixation duration is one of the most commonly used metrics obtained by eye-tracking devices and is also one of the most typical indicators for measuring participants’ attention allocation during the eye movement process (Duque and Vázquez, 2015). The length of fixation duration represents readers’ attention to the information in observation area and the depth of cognitive processing (Yu et al., 2018; Wolf et al., 2019). A longer fixation duration indicates that participants find the information in that area more difficult to process or that the information in that area attracts them more (Wu and Liu, 2019).

**Figure 3:**
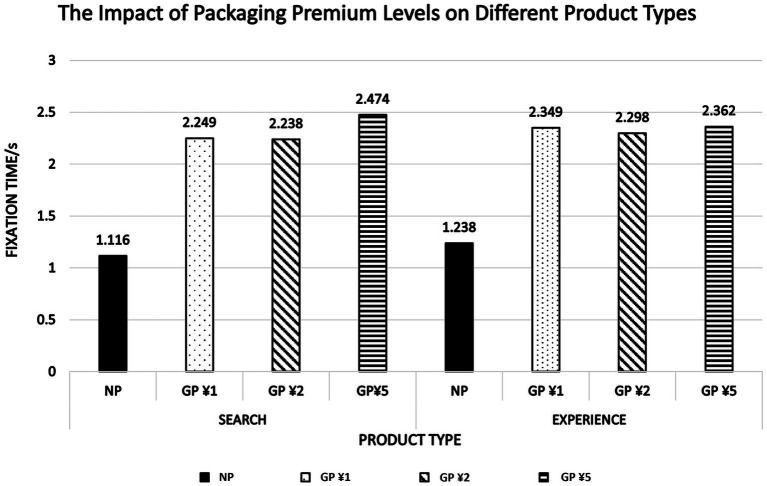
Packaging premium levels and product types analysis.

In this experiment, the average fixation time of 59 participants browsing two different product checkout pages was statistically analyzed, and the descriptive statistics are shown in Figure 3. For search products, the fixation time for the green express option group (*M*_1_ = 2.249, SE = 1.111; *M*_2_ = 2.238, SE = 1.113; *M*_5_ = 2.474, SE = 1.084) was significantly higher than that of the regular express option group (*M*_0_ = 1.116, SE = 0.614). For experience products, the fixation time for the green express option group (*M*_1_ = 2.349, SE = 1.454; *M*_2_ = 2.298, SE = 1.445; *M*_5_ = 2.362, SE = 0.983) was significantly higher than that of the regular express option group (*M*_0_ = 1.238, SE = 0.745). This result indicates that participants paid more attention when facing green express options. H1 is verified.

A mixed repeated measures ANOVA was conducted on the fixation duration of the green express interest area for both product types. The results show that the main effect of product type was not significant (*F*(1,219) = 0.204, *p* = 0.652 > 0.050, *η*^2^ = 0.001), meaning there was no significant difference in the fixation time on the interest areas for express options of search products and experience products. The main effect of premium level was significant (*F*(3,219) = 25.467, *p* = < 0.001, *η*^2^ = 0.259), with the fixation time for the green express options at low, medium, and high premium levels for both product types being significantly higher than that for the regular express options with no premium. The interaction effect between product type and premium level was not significant (*F*(3,219) = 0.321, *p* = 0.810 > 0.050, *η*^2^ = 0.004). The data show that fixation duration on green express options is primarily driven by the premium levels, with longer fixation for both sorts of products as the premium increases. The lack of the significant interaction between product type and premium level suggests that product type does not influence visual attention to GEP. Therefore, consumers’ fixation duration on the green express option is significantly influenced by the premium level but is not moderated by product type or its interaction with the premium. H2 was not supported.

### 4.4 Eye-tracking heatmap analysis

**Figure 4:**
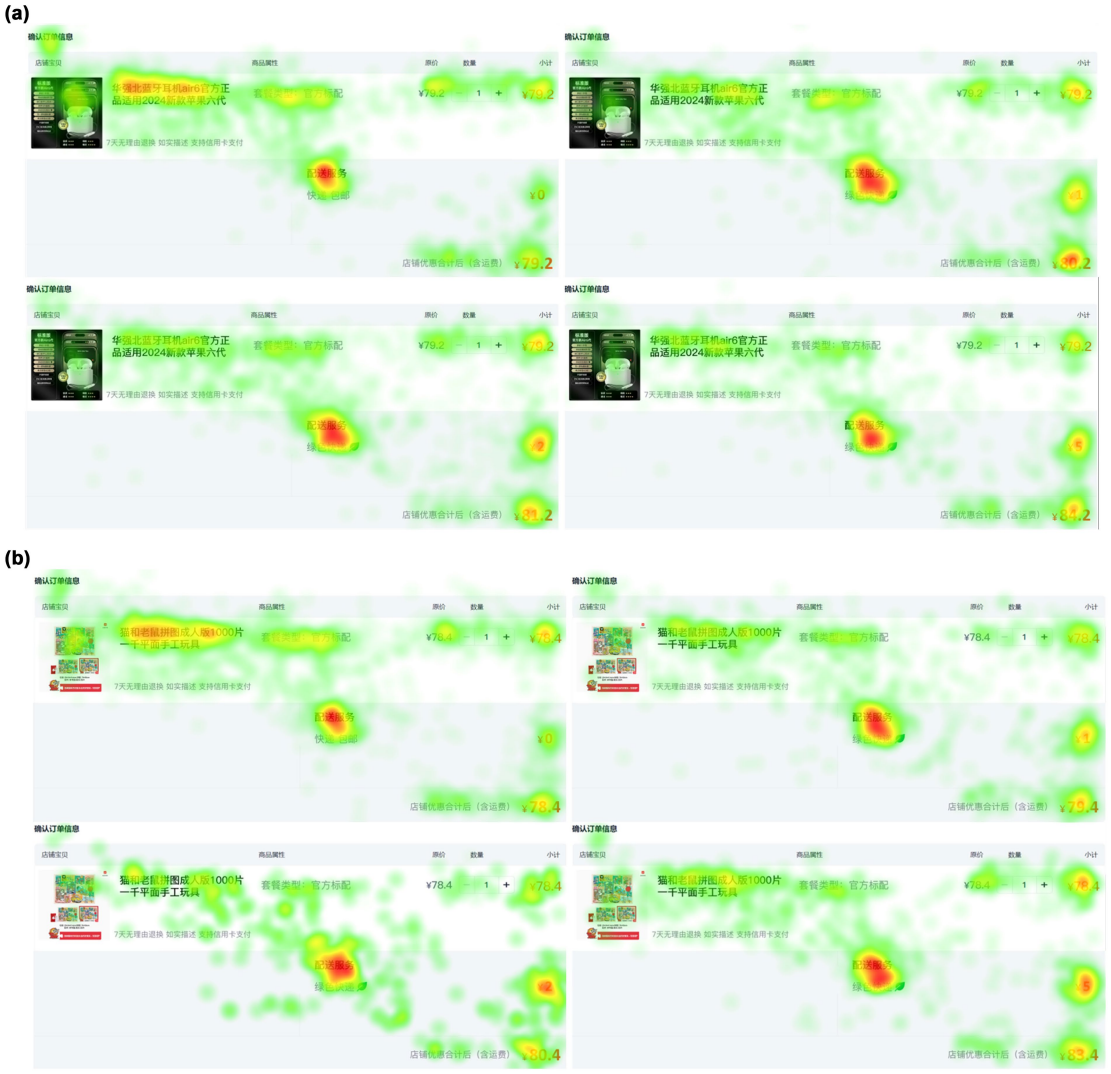
Heatmap of the checkout interface AOI for different product types. **(a)** Search products. **(b)** Experience products.

The heatmap provides an intuitive reflection of consumers’ attention to the observed area. The deeper the color, approaching red, the longer and more frequent the consumer’s attention, followed by yellow, with green indicating the least attention (Zhen et al., 2024). Figure 4 presents a comparative analysis of heatmaps for the areas of interest in the checkout pages of two types of products: (Figure 4a) heatmap of search products (Bluetooth headphones), and (Figure 4b) heatmap of experience products (adult puzzles). In these figures, the express prices, from left to right, are 0, 1, 2, and 5 yuan. By comparing the color differences between Figures 4a, b it is clear that consumers’ attention to the green express option is significantly higher than that for traditional express methods. When facing search products, consumers’ attention to different premium levels increases with the price, especially in the ¥1 and ¥2 premium levels. However, when faced with a ¥5 premium, consumer attention significantly decreases, possibly because the price exceeded their psychological expectation, causing them to automatically disregard the higher-priced option. In contrast, for experience products, consumers’ attention to the premium price increased as the price rose, indicating that even with higher prices, consumers were willing to spend more time considering the environmental attributes. The heatmap differences between the two product types further suggest that consumers weigh environmental value against price cost more heavily when purchasing experience products, whereas they are more price-sensitive when purchasing search products. Consequently, H1 is directly verified by the heatmap analysis. H3 is partially verified, as the high premium for green express reduced participants’ attention to search products, but did not reduce attention to experience products.

## 5 Conclusion

This paper combines questionnaire survey and eye-tracking experiment to explore the influencing mechanisms of consumers’ willingness to pay for GEP. The following conclusions are drawn.

There is a significant positive relationship between consumers’ environmental awareness and their willingness to pay a GEP premium. Specifically, consumers with higher environmental awareness are more likely to choose green express when faced with a premium, and they are willing to pay a higher premium for it. This finding contrasts with previous studies that primarily relied on subjective questionnaire data (Zhao and Han, 2015; Wang et al., 2016), which reflected consumers’ self-reported preferences and intentions rather than actual behavioral choices.

Consumers’ willingness to pay is not only influenced by environmental awareness but also moderated by the type of products. Consumers’ willingness to pay for green express is generally higher for experience products than for search products, indicating that products driven by emotional value are more likely to motivate consumers to pay for environmental attributes.

Price factor plays an important restrictive role in GEP consumption decision. Under different premium levels, both low and high environmental awareness groups show the highest choice rate when faced with low premiums (¥1–¥2). As the premium level increases, the selection rate for green express gradually decreases. Objective eye tracking data not only validates Hao’s findings on the factors influencing consumers’ willingness to pay for green products, but also supports the average value of GEP premium by Lin and Jia et al.

The eye-tracking experiment further reveals the distribution of consumers’ visual attention when choosing GEP. Consumers with higher environmental awareness spend significantly more time and attention when facing the green express option, reflecting their high level of concern for environmental information, and they are more inclined to choose GEP in the decision-making process. Heatmap and fixation duration analyses in the experiment also indicate that the green express option attracts more visual attention than the regular express option, especially when faced with a higher premium. Although the willingness to pay decreases, GEP still receives higher attention.

The hypothesis validation results of this paper indicate that: H1 (consumers have a higher willingness to pay for GEP of experience goods compared to search goods) was supported; H2 (product type moderates fixation duration on green express options) was not supported, as fixation duration was primarily driven by premium levels; H3 (the interaction effect between premium level and product type on willingness to pay) was partially supported, with high premiums suppressing the selection rate of search goods more prominently, but the interaction effect was statistically insignificant; H4 (higher environmental awareness positively influences willingness to pay for GEP) was supported, yet its effect was significantly constrained by premium levels.

## 6 Implications

### 6.1 Theoretical implications

This paper makes several significant theoretical contributions to the understanding of consumer behavior in the context of e-commerce, particularly in relation to GEP and its willingness to pay. First, it extends the theory of planned behavior (TPB) by integrating environmental awareness as a key factor influencing consumer decision-making in the context of GEP. TPB has been used to explore the impact of online reviews on consumers’ green purchase intentions (Zhang et al., 2023). This paper emphasizes the role of environmental responsibility and its impact on consumers’ willingness to pay for GEP. The empirical findings suggest that environmental awareness significantly drives consumers’ green consumption behaviors, enriching the TPB framework by highlighting the influence of ecological values on consumer choices in e-commerce.

Another innovative theoretical contribution of this paper is the integration of eye-tracking technology to explore how consumers’ visual attention influences their decision-making process when selecting green express options. Some studies have provided important insights into how visual elements (such as price displays and green express) influence consumer preferences and purchase intentions, but these have primarily relied on survey-based methods (Chen et al., 2024). Eye-tracking technology has also been used to explore consumers’ purchase intentions in physical retail environments (Clement et al., 2015; Wästlund et al., 2018). So the use of eye-tracking provides a more objective and detailed insight into consumer attention allocation online. The results from this study suggest that eye-tracking metrics (such as fixation duration and attention span) can be powerful indicators of consumer preferences, particularly when examining how consumers evaluate GEP options at different price points. This method offers a valuable tool for future research that aims to investigate consumer behavior in real-time.

Finally, this paper contributes to the field of visual marketing by exploring consumers’ willingness to pay a premium for green express options. Previous research on visual marketing has mainly focused on advertising design (Higgins et al., 2014), food packaging design and product appearance (Husić-Mehmedović et al., 2017), with limited attention given to the elements of product checkout pages. This study highlights the impact of GEP options and pricing elements on consumers’ visual attention, providing valuable insights for e-commerce platform web designers.

### 6.2 Practical implications

This paper offers practical implications for e-commerce platforms and express companies, focusing on strategies for differentiated pricing and interface design optimization. For experience products, e-commerce platforms should allow a moderate GEP premiums for them, where consumers show a higher willingness to pay. Data suggested that the optimal premium should range from ¥1 to ¥2, as consumers’ selection rate for green express is highest in this price range. This can be communicated through visual cues emphasizing the environmental benefits of these products; for search products, consumers are more price-sensitive, stricter premium control should be enforced. Offering lower or zero additional charges for GEP will be important to maintaining a competitive advantage and appealing to eco-conscious consumers without deterring them due to higher prices.

Eye tracking data indicates that consumers with higher environmental awareness spend significantly more time and attention on green express selection. E-commerce platforms can personalize green promotional messages to promote green express application by data-driven user profiling, offering targeted recommendations based on consumers’ shopping behavior and environmental awareness. Dynamic images or environmental benefit icons can be used to attract more attention from consumers (Wang, 2023) while optimized the product checkout interface or express delivery website to increase the likelihood of green express delivery selection.

### 6.3 Limitations and future research

Although this study provides an analysis of college students’ willingness to pay for GEP, it still has some limitations. First, the sample size is limited to students from a single university. Future research should consider expanding the sample mount and scope to include consumers from different age groups, professions, and income levels to improve the generalizability of the findings. Second, the premium levels in the experimental design may not fully simulate the complexity of real-world shopping scenarios. Future studies could explore consumer behavior in more realistic contexts. Furthermore, while eye-tracking data showed increased attention to green express options, but we did not explore the nature of this attention. It is unclear whether it is driven by positive evaluation, novelty, or cognitive difficulty. Future research should examine these factors to better understand consumers’ motivations. Studies could incorporate emotion recognition or cognitive load assessments to explore how these factors influence attention and decision-making.

## Data Availability

The raw data supporting the conclusions of this article will be made available by the authors, without undue reservation.
